# To Raise or Not to Raise the Level of Ingredients in Yoghurts: Polish Consumer Preferences Regarding Dairy Products

**DOI:** 10.3390/nu11102526

**Published:** 2019-10-19

**Authors:** Marta Sajdakowska, Agnieszka Tekień

**Affiliations:** Department of Food Market and Consumer Research, Institute of Human Nutrition Sciences, Warsaw University of Life Sciences (SGGW-WULS), 159C Nowoursynowska Street, 02-787 Warsaw, Poland; agnieszka_tekien@sggw.pl

**Keywords:** consumer, yoghurt, label, a discrete choice-based experiment

## Abstract

Modern consumers are becoming increasingly aware of the perceived health benefits of food. As a result, they are in search of various types of information, for example, information on the packaging of food products that could confirm to what extent the purchased product will meet their expectations regarding the proper composition, that is, nutritional value, or perceived health values earlier mentioned. Furthermore, consumers increasingly seek new dairy products with additional health benefits and, therefore, it is essential to explore which attributes are important drivers of food choices and how producers can better respond to shifting consumer values and needs in each dairy product category. Therefore, the aims of our research was twofold: (1) To determine different segments of consumers based on their preferences towards food and nutrition, including opinion on new food products with a particular emphasis on a dairy market as well as (2) to study the importance of some statements related to nutrition presented on the yoghurt label with a precise focus on aspects of the increased and decreased content of some ingredients. The data were collected using a CAPI (Computer Assisted Personal Interview) survey on a sample of 489 adult Polish consumers. Respondents provided answers to questions and took part in a discrete choice-based experiment. The obtained data were analysed using the clustering method. The segmentation was performed using a hierarchical Ward’s method. As a result, four segments were identified: Quality-oriented, Involved, Quality Enthusiasts, and Neutral. The results indicated that in relation to the features that are important in the case of yoghurts, the following were indicated above all: Beneficial effects on health, its sensory values, as well as its availability on the market and production by traditional methods. Consumers belonging to Quality Enthusiasts seemed to be the most promising segment due to their openness to new products, as well as positive feedback on yoghurt. From the perspective of taking action on the food market, Involved may also be interesting, as it showed their openness to new products available on the food market. However, due to the relatively lower, compared to other segments, assessment on the beneficial effect of yoghurt on health, their taste, aroma, availability, as well as the importance of information on care for the proper method of breeding animals, this segment can pose a special challenge to entrepreneurs. Moreover, Involved seemed to be more demanding and critical towards some projects undertaken on the market by policy makers and marketing practitioners.

## 1. Introduction

In the last years, consumer demand for health-enhancing food products has risen rapidly [1,2,3,4]. On the one hand, the changing lifestyles and growing health concerns towards the negative impact of certain food products, e.g., saturated fatty acids originating from animals has increased consumer preferences for dairy-alternative products [5].

On the other hand, the review of available scientific evidence conducted by Thorning et al. (2016) supported the fact that the intake of milk and dairy products contributes to meeting nutrient recommendations and may protect against the most prevalent, chronic non-communicable diseases [3]. Tapsell (2015) stated that a combination of evidence is still necessary and more research is needed across various regions, but indications remain that fermented dairy foods such as yoghurt and cheese are an integral part of diets that are protective against cardiovascular disease (CVD) [4].

Results of other studies indicate that the suggestion to restrict or eliminate full-fat dairy from the diet may not be the optimal strategy for reducing cardiometabolic disease risk and should be re-evaluated in light of recent evidence [6].

Results of studies of Mazidi et al. (2019) showed that higher total dairy consumption was associated with lower total and cerebrovascular mortality, while higher milk consumption was associated with higher risk of coronary heart diseases (CHD). These results do not support public health advice to reduce total dairy fat consumption, although the association between milk consumption and CHD mortality requires further study [7].

Moreover, there is evidence for the impact of fermented foods and beverages (e.g., yoghurt), produced or preserved by the action of microorganisms, on general health, namely their significance on the gut microbiota balance and brain functionality [2].

In addition to this, when it comes to yoghurt, it is the most frequently consumed healthy and nutritious food around the world. Therefore, it has potential in conveying nutritious ingredients to the human diet. Most people in developing or underdeveloped countries suffer from micronutrient deficiencies and enriched food products can reduce nutritional diseases. A study shows that food enrichment can prevent most diseases and it has a significant impact in improving the health of the community [8]. Furthermore, yoghurt consumption is associated with a lower body mass index, lower body weight, smaller waist circumference, and lower body fat in epidemiological studies [9]. Moreover, one should also not forget about the indirect economic benefits associated with the consumption of yoghurt. Increasing yoghurt consumption in the adult population in the UK by 100g per day could generate substantial cost savings to the UK National Health Service as well as significant benefits for a patient through reductions in the incidence of type 2 diabetes (T2D) [10].

Apart from the aspect related to the beneficial effects of food on health, information on the product’s label also plays a major role in consumer decisions [11]. Moreover, there is some empirical evidence for the existence of a positive relationship between nutritional label use and healthy food choices [12]. In general, food labelling is regulated in order to both help consumers make informed choices regarding the food they consume and to prevent any practices that may mislead them [13]. Furthermore, differences in nutritional knowledge related to nutritional recommendations and the links between nutrient consumption and health have been the main reason for differences in healthiness perception and willingness to try functional foods among consumers [14]. With regards to yoghurt, sweetness and information on sugar content have had significant effects on liking and purchase probability [15].

Other research findings showed that the response to labels differ, both with regard to the way information is presented (facts or claims) and with the type of information (nutrition or health) offered. Consumer utility increases when nutrition and health information labels in food products are present and tends to provide higher utility than facts panel only in the case of the less healthy product [16]. While consumers generally consider the nutrition composition of yoghurt to be more important than the tested claims, some groups of consumers are more sensitive to the use of health-related statements [13].

Since some consumers do not understand health and nutrition claims and even express doubts over the claimed effects of food, the importance of information about the content of a nutrient in the product should also be examined in order to find out which attribute is more convincing for consumers when the purchase decisions are being made [17].

Apart from the health-related aspects of dairy products and the role of food information labels in consumer purchasing decisions, generally, consumer knowledge of the relationship between diet and health is important for innovation in the food sector, therefore, producers must find a way to reduce unhealthy ingredients and enhance healthy ones in food products [18].

Producers have focused on reducing sodium, fat, cholesterol contents in food products [19] as well as in reducing sugar contents, and even some recommendations have been proposed by policy makers [20].

Therefore, the aims of our research was in twofold: (1) To determine different segments of consumers based on their preferences towards food and nutrition including opinion on new food products with a particular emphasis on the dairy market as well as (2) to study the importance of some statements related to nutrition presented on the yoghurt label with a precise focus on aspects of the increased and decreased content of some ingredients.

## 2. Material and Methods

### 2.1. Design of the Experiment

In our research, a discrete choice-based experiment was conducted in order to elicit consumer preferences referring to different product profiles with some levels of attributes [21], so study participants made a discrete choice from a set of presented alternatives which contained a number of attributes with different levels, combined within choice sets [22]. It was analysed with discrete choice models [23] in order to get utilities important to respondents from a situation where consumers choose a particular product among available products [24]. This kind of experiment was selected instead of rating-based conjoint due to its greater similarity with market behaviour, i.e., more similar to what a consumer really does when buying food [25].

### 2.2. Data Collection Process

**Figure 1:**
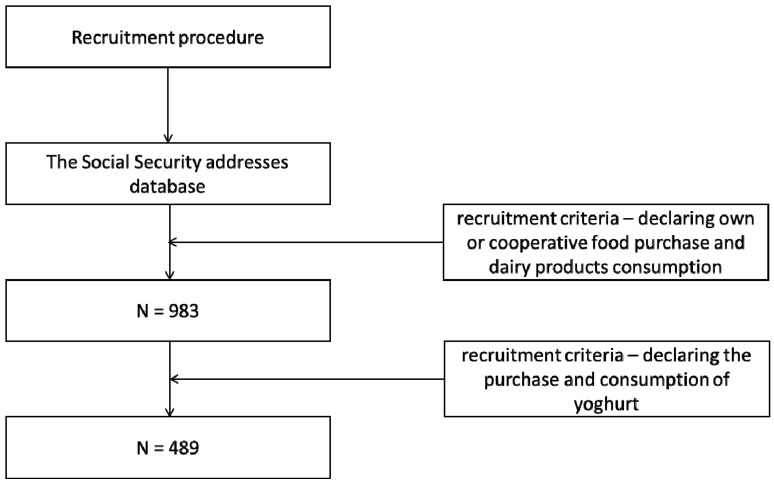
Participants’ inclusion in the study.

This paper presents some of the findings from a larger study [26]. The sample was drawn from the Social Security addresses database and was representative in terms of age and gender. The survey was conducted in each of the 16 voivodships in Poland. After drawing the starting addresses, the method of random route was used in the selection of the sample [27]. The interviews were conducted face-to-face at respondents’ homes by a professional market research agency, respecting the ESOMAR (European Society for Opinion and Marketing Research) code of conduct using the CAPI (Computer Assisted Personal Interview) technique. All respondents were aged 21 years and over. Only respondents who met the recruitment criteria, i.e., made their own or cooperative food purchase and declared dairy products consumption, participated in the study. Those who declared the purchase and consumption of yoghurts among consumers from the total sample, took part in the research (*N* = 489) (Figure 1). 

### 2.3. Consumer Attributes

Taking into account various determinants that are important for consumers during the purchasing choice of yoghurt [25], we decided to select the following for the experiment attributes: Increased content of ingredients (8 levels), reduced content of ingredients (5 levels), additional claims (4 levels), and price (12 levels). Attributes and their levels are presented in Table 1.

During testing, the respondents were presented with screens displaying the full characteristics of each product (every product was described on each of the attributes). As shown in Table 2, 3 product configurations were presented on the screen and the respondent had to indicate the preferred alternative. In this study, a “no-choice” option was not included although it has been pointed out that sometimes this option may give a better market penetration prediction [23]. As a result, it was a situation of a forced choice where the respondent had to opt for one configuration in order to choose the preferred product (out of 3) to buy. Each respondent had to choose one out of 3 products. The task was repeated 12 times for each respondent. During the research, consumers were asked the following question: “Which yoghurt do you want to buy?” 

### 2.4. Data Analysis

The Hierarchical Bayesian (HB) network model was applied in Sawtooth SMRT (Sawtooth Software Market Research Tool). This is a dedicated software for a discrete choice-based experiment, which enables the estimation of coefficients for the individual utilities of each attribute level as well as an estimate of individual-level part-worth values. The HB algorithm has the ability to borrow information from other respondents in order to stabilise part-worth estimation for each individual. It is a valuable feature for this method. The hierarchy of the Bayesian network results from the fact that two levels can be distinguished [24]. At a higher level, the assumption is that each individual’s part-worths are described by a multivariate normal distribution. Such a distribution is characterised by a vector of means and a covariance matrix. At a lower level, it is assumed that given the individual’s part-worths, their probabilities of choosing particular alternatives are governed by a multinomial logit model [28]. After modelling with the HB network, part-worth utilities for all product attributes and levels were obtained and, in further studies, an analysis in IBM SPSS Statistics version 23PL (IBM Corp. in Armonk, NY, USA) was carried out to study the data more closely. The script for this discrete choice-based experiment was prepared in the Sawtooth SMRT software v. 4.22 (Sawtooth Software, Provo, UT, USA). A “full profile” option was used as a rotation scheme of used product variants. This means that all of the attributes of each product were always shown on screen. The profiles were generated using the complete enumeration method, where each presented product profile differed from the adjacent product in each of the presented attributes.

In order to obtain an in-depth analysis, the sample of respondents who took part in the discrete choice-based study was subjected to segmentation. The following questions from the survey questionnaire were selected as the basis for segmentation: (1) Please specify how much you agree or disagree with the following statements: “I like to buy new dairy products”, where 1 means I definitely disagree and 7 means I definitely agree, (2) “How important is it for you that the yoghurt you eat does not contain artificial additives”, where 1 means definitely not important and 7 means definitely important, as well as (3) “How important is it for you that the yoghurt you eat is organic”, where 1 means definitely not important and 7 means definitely important.

Segmentation was performed in SPSS v.23 using the hierarchical Ward’s method. Using Ward’s method [28,29] for each cluster, the means were calculated for all variables. In the next step, the squared Euclidean distance to the cluster means was calculated for each considered case. Then, all these distances were summed for all of the cases. At each step, the two clusters to be merged were those that resulted in the smallest increase in the overall sum of the squared within-cluster distances. The coefficient in the agglomeration schedule is the within-cluster sum of squares at that step. It is not the distance at which clusters are joined. 

## 3. Results

### 3.1. Socio-Demographic Profile of the Total Sample and the Clusters Identified

As a result of the segmentation analysis, four cardinality clusters (segments) were identified from the following multiplicity: Cluster 1 (*N* = 78, 16% of total sample), cluster 2 (*N* = 123, 25%), cluster 3 (*N* = 233, 48%), and cluster 4 (*N* = 55, 11%) (Table 3). In the next step, the data from the discrete choice-based experiment module was calculated for all the clusters.

In the examined general sample (Table 3), women dominated (59%). Taking age into account, it shows that the majority of respondents were aged 35–54 years and up to 34 (39% and 37%, respectively). Over 2/5 of respondents (41%) had secondary education and almost 1/3 had vocational education (29%). Taking into account the place of residence indicates that over 1/3 of respondents (34%) live in the country, every fifth respondent live in cities with a population of 20,000–100,000 inhabitants (21%), and cities of 100,000–500,000 inhabitants (18%). Almost half of the respondents declared having children (49%) and almost 70% of the surveyed assessed that they lived frugally and had enough money to buy what they needed, and that they lived very frugally to save money for major purchases (40% and 28%, respectively). In segment 1 (16%), men (53%) dominated as well as people aged 35–54 years (40%) as well as people with a secondary (38%) and higher education level (26%), live in the country (37%) and cities of 20,000–100,000 residents (26%), and have children (46%). Almost half of the respondents in segment 1 declared that they lived frugally and had enough money for everything (47%). In segment 2 (25%), women (58%) dominated as well as those up to 34 years and those aged 35–54 years (40% and 37%, respectively), have secondary education (46%), reside in a village and town of 20,000–100,000 residents respectively (29% and 24%). Almost half of the people in this segment declared that they have children (46%), and more than 2/5 said that they live frugally and have enough money for everything (41%). In segment 3 (48%), men (63%), people up to 34 years of age and 35–54 years (43% and 37%, respectively), as well as people with secondary education (43%) dominated. Segment 3 comprised of people living in the country and large cities of 100,000–500,000 inhabitants (29% and 24% respectively), have children (48%), and declare that they live frugally and have enough for everything (38%), and that they live very frugally to save money for major purchases (31%).

In segment 4 (11%), women (56%), people aged 35–54 years (53%), live in the country (47%), and have children (62%) dominated. Similarly to segment 3, those belonging to segment 4 declared that they live frugally and have money for everything (38%) or that they live very frugally to save money for major purchases (31%) (Table 3).

In order to characterise the segments, several questions from the questionnaire were used. The first (1) of them concerned opinions on the quality of dairy products: “Please indicate how much you agree with the following statements”. Please provide answers on a scale of 1–7, where 1 meant “definitely disagree” and 7 meant “definitely agree”. Agreement was assessed in relation to the following statements: Quality is important to me when choosing dairy products, I buy high-quality dairy products because they have a good effect on my children’s health, I buy high-quality dairy products because they have a positive effect on my body shape, I buy high-quality dairy products for family members who have health issues. These statements referring to the importance of food quality were also used as the basis for naming individual segments: Quality-oriented (cluster 1), Involved (cluster 2), Quality Enthusiasts (cluster 3), and Neutral (cluster 4) (Table 4).

The next questions that were used in segment profiling were: (2) “Please say how much you agree or disagree with the statements about dairy products”, where 1 meant strongly disagree and 7 meant strongly agree and they referred to new dairy products available on the market. Table 4 presents the items used in the question. The next question concerned selected features of yoghurts and was: (3) “Which of the following features of yoghurts which you usually consume are important for you?”, where 1 meant definitely not important and 7 meant definitely important. The last question that was used in profiling (4) related to information that refers to the presence of various ingredients in yoghurts and was: “Please specify if you would be interested in the following types of yoghurts”. Please use a scale from 1–7, where 1 meant definitely not and 7 meant definitely yes.

Analysis of the data presented in Table 4 indicates that the quality of dairy products played a major role in the purchasing decisions of the respondents. This thesis is confirmed by relatively high levels of agreement with the statement: “Quality is important to me when choosing dairy products”. The respondents also declared a high level of agreement with the statement: “I buy high-quality dairy products because they have a good impact on the health of my children” (5.11), but no differences at a statistically significant level were found between individual clusters. At a medium level, there was an agreement with the statement: “I buy high-quality dairy products because they have a positive effect on my body shape”, while respondents in clusters 2 and 3 appreciated the impact on the body shape to be significantly higher compared to respondents in segments 1 and 4.

Regarding the agreement with statements on openness to buy new products and changes in the market including dairy products, the respondents agreed most strongly with the following statements: “I am very particular about the new dairy products I will eat (4.79)”, “Ethnic food deprived of dairy products looks too weird to eat (e.g., Asian cuisine)” (4.58), and “If I do not know what is in a dairy product, I will not try it” (4.54).

In relation to the above statements, no statistically significant differences were noted between individual segments. Further statements about new dairy products with average scores of 4 and above, i.e., “New dairy products arouse my curiosity” (4.39), “I trust new dairy products”, “I like to buy new and various dairy products” (4.26), “I will eat virtually everything” (4.16), and “At dinner parties, I will try new dishes based on dairy products” (4.09) had a significantly higher level of agreement in segments 2 and 3 compared to segments 1 and 4, which may indicate a greater openness of consumers in segments 2 and 3 to new products on the food/dairy market compared to those in segments 1 and 4.

### 3.2. Attitudinal Questionnaire

Analysis of the data presented in Table 5 indicates that in relation to the features that are important in the case of yoghurts, the following were indicated above all: Beneficial effects on health, its sensory values (statements about taste and smell), as well as its availability on the market and production by traditional methods. Furthermore, the Polish origin of yoghurt and the origin of farms where the producer cares in a special way for the proper way of breeding animals. Respondents from segment 2 assessed the following aspects lower than in other segments: The significance of beneficial effects on health, flavour, availability of yoghurts, and information on Polish origin. Compared to other segments, these surveyed features also showed the least appreciation for the importance of the origin of yoghurt from farms where the producer pays special attention to the proper way of breeding animals. 

Analysis of the data presented in Table 6 indicates that in relation to specific examples of information that would accompany the availability of yoghurt on the market, the respondents most often declared interest in yoghurt “with live bacteria cultures” and “produced using raw materials from organic farms”. Next, interest was indicated in yoghurts with increased levels of substances beneficial to health with reduced levels of some ingredients, e.g., salt or sugar, in order to prevent various diseases, e.g., obesity, hypertension, diabetes, followed by yoghurts with higher levels of certain vitamins and minerals in order to prevent nutritional deficiencies. It was noted that respondents in segments 1 and 3 were significantly more interested in yoghurts with live bacteria cultures and yoghurts produced using raw materials from organic farms than those in segments 2 and 4. Respondents from segment 3 obtained the highest average scores compared to other segments in relation to yoghurts with live bacteria cultures with increased levels of substances beneficial to health, yoghurts with higher levels of some vitamins and minerals, and with reduced levels of some ingredients, e.g., salt or sugar, to prevent various diseases such as obesity, hypertension, and diabetes. On the other hand, respondents from segment 1 compared to other surveyed features obtained the highest rating in relation to yoghurts produced using raw materials from organic farms. In the case of segment 2, relatively low scores were obtained in comparison to other segments with regard to yoghurts with live bacteria cultures made from raw materials from organic farms with reduced levels of some ingredients, e.g., salt or sugar, in order to prevent various diseases such as obesity, hypertension, and diabetes, as well as an increased level of substances with beneficial effects on health.

As indicated in the previous part of the study, in the next part of the survey the respondents were asked to answer the question: “Which yogurt would you like to buy?” using the discrete choice-based experiment method. The subjects were offered several attributes of yogurt: (1) Increased level of ingredients, (2) reduced level of ingredients, (3) additional information, and (4) price.

### 3.3. The Relative Importance of Attributes

An analysis of the values (mean relatives) presented in Table 7 indicates that consumers perceived the price and information on increasing selected ingredients in yoghurts as the two most important attributes prompting them to buy food products/yoghurts (70.4% and 15.7%, respectively), and the following “additional information” and information on reducing selected ingredients (8.5% and 5.4%, respectively) were mentioned. Along with the increase in price, a decrease in utility/lower utility was noted. Regarding ingredients with increased levels, the most accepted information was “With an increased number of live bacteria cultures”, “With an increased amount of vitamins and minerals”, and “With an increased amount of fiber” (0.939, 0.546, 0.453, respectively). 

With regard to “additional information”, the highest level of preferences was noted for information: On high health values (0.315), weight is attached to the role of health in general compared to information on high nutritional value and high quality (0.257 and 0.155, respectively). When the information was not available (No information), negative usability was noted (−0.727). 

For information relating to reduced levels of ingredients: A higher level of utility was obtained for the information “No added sugar” (0.332) compared to the information “Reduced sugar” (0.270). Other levels of attributes have negative usability. For respondents in segment 1, the most important factors taken into account when choosing yoghurt was information on ingredients whose amount in the product was increased (19.4%) and information on ingredients whose amount was reduced (8.5%). On the other hand, the significance of price was the lowest, compared to the opinions of people in other segments (63.7%). For consumers in segment 2, the price was the most important factor during the purchase of yoghurt (75.4%) compared to the sequence of factors for other segments and the remaining attributes played a relatively less significant role. In the case of segment 3, respondents attached a lot of importance to “additional information” compared to other segments (9.2%). The significance of the price was relatively lower for segment 3 (68.7%) compared to segment 2 (75.4%), as well as compared to segment 4 (71.9%). As noted, the respondents in segment 4 belonged to the group of people for whom the price was relatively important, but they also attached attention to information on increasing selected components (16.3%). For both segment 1 and segment 4, for ingredients whose increase was most preferred, the highest part-worth utilities were recorded for information “With increased number of live bacteria cultures” (1.160 and 1.209, respectively).

## 4. Discussion

The study presents the results of a consumer survey using a questionnaire among Polish consumers. The analysis of the results obtained indicated that the quality of dairy products is of great importance to Polish consumers. This is confirmed by the results of studies by other authors, in which it was emphasised that the perception of food quality by consumers affect their purchase decisions and dietary patterns [29]. In addition, the survey found that some consumers were open to food and nutrition news. This concerned two consumer segments (2 and 3). These two segments compared to 1 and 4 also contained, apart from those aged over 35, relatively more younger consumers, i.e., 34 and below, which may have affected the acceptance of new/innovative products as indicated by the results of other surveys among consumers, including those relating to Polish consumers [30].

### 4.1. The Most Important Yoghurt Features

Regarding yoghurts, among the most important features characterising them, beneficial effects on health, flavour, and availability on the market were indicated. The literature confirms the obtained research results refering to flavour and availability [15,31,32], as well as the importance of the perceived beneficial impact of food on health was indicated in other consumer studies [26,30,33,34,35,36].

Moreover, as consumers demand healthy foods with a pleasant taste, in recent years, some functional dairy products have been produced by means of enrichment and fortification. Consequently, yoghurt has begun to attract new consumer groups due to its pleasant taste and increased health benefits [37].

The beneficial effect on health in the described own study was also confirmed by relatively high assessments regarding the presence of certain ingredients in food. Augmenting the ingredients whose increased quantity positively associates with health effects e.g., fiber, vitamins, and minerals, was strongly accepted and the reduced amount of ingredients that consumers perceive as negatively affecting health e.g., sugar or fat, was accepted. This is confirmed by the acceptance of changes in food of animal origin by Polish consumers [38]. In addition, the literature indicates that for individual ingredients that have been raised or lowered, some consumers accept fiber-enriched yoghurt [39]. Moreover, when it comes to vitamins and minerals, due to the high consumption rate of dairy products such as yoghurt, the fortification of these products will effectively reduce or prevent diseases associated with nutritional deficiencies [8]. When it comes to reducing the level of sugar and fat in dairy products, the results of research among Polish consumers confirm the preferences in this category of products [38].

In the case of this research, the method of producing yoghurt is also worth emphasising, in particular referring to traditional production methods and involvement in animal welfare. These two aspects are particularly important because of the perceived quality of dairy products by consumers, including their freshness and taste [31,40,41,42]. This is also confirmed by the research of other authors of the source, in which attention is drawn to the importance of traditional production methods [43] and the growing importance of caring for animals in consumer statements [44,45]. However, the importance of the aspect related to caring for animals is presented differently, depending on the study [46,47].

The discrete choice-based study also noted the importance of price as a factor in food, which is confirmed by the studies of other authors [33,48,49,50]. Price was ranked as the most important food choice factor in five countries (Spain, Greece, Ireland, Portugal, and the Netherlands), sensory appeal was ranked first for three countries (Norway, Germany, and the UK), while natural content was ranked as the most important factor in Poland. Familiarity and ethical concern were consistently ranked as the least important in all countries [51]. The results of the review made by Roman et al. (2017) clearly showed that for the majority of consumers in developed countries, naturalness in food products is important. This finding could be observed across countries and in the different years when the studies were conducted [52]. On the other hand, Aschemann-Witzel [53] indicated that the consumer’s role and consumption aspect of the supply chain were identified to be crucial in improving healthy choices and achieving sustainability goals. 

As for the importance of live bacteria cultures, it was also important for the subjects tested. This may be associated with the generally positive perception of yoghurt through the prism of the content of live bacterial cultures, which is confirmed by the studies of other authors [54,55]. Research shows that yoghurt still plays an important role in the human diet today due to its pleasant taste and health benefits. To meet consumer demands for healthier options, manufacturers are making low fat and non-fat versions of their most popular flavours. Unfortunately, when adding prebiotics to yoghurt, a negative impact was observed on the sensory characteristics of the yoghurt turning off consumers [37].

### 4.2. Perspectives of Information on the Yoghurt‘s Labels

Although the health motive seems to be one of the most important factors affecting functional foods, its effect partially depends on the consumer’s knowledge of a particular health-enhancing effect, as well as on the barriers and benefits they perceive from the use of nutritional labels [56]. In general, nutritional claim (NC) requirements on food packages are among the most important and influential EU policy measures related to diet, which has the capacity to promote healthy eating. The results of other research indicate that the low-sugar NC was the least preferred claim. Overall, the presence of NCs generally increases visual attention, which may be linked to an increased likelihood of affecting the final decision to purchase yogurts with NCs [57]. In summary, yoghurt has always been one of the vital players in the spectrum of fermented food products that has transformed science and technology into health and wellness through diet. Considering the fast evolution of functional yoghurts either at a research stage or marketplace, further development should demand an accurate measurement of quality, safety, and efficacy to meet consumer expectations on quality and claimable health benefits [54]. Studies show that the acceptance of products with health claims is influenced by several factors. Familiarity with the product, health claim, or functional ingredient used plus personal relevance appear as the most important determinants. The choice of a carrier product can determine to what extent people trust a health claim or are willing to try the respective product. Furthermore, consumers like simple wording, but they may also demand detailed explanations. However, more research is needed on consumer understanding of health claims in order to maximise the potential for functional foods in order to contribute to healthy, balanced diets [58].

## 5. Conclusions

An analysis of the available results of our research indicated that consumers belonging to segment 3 (Quality Enthusiasts) seemed to be the most promising segment due to their openness to new products, as well as positive feedback on yoghurt. From the perspective of taking action on the food market, segment 2 (Involved) may also be interesting, as it showed openness to new products available on the food market. However, due to the relatively low, compared to other segments, assessment on the beneficial effect of yoghurt on health, their taste and aroma, availability, as well as the importance of information on the proper way of breeding animals, this segment could be a special challenge for entrepreneurs. Segment 2 seemed to be more demanding and critical (or perhaps more cautious) regarding some projects undertaken on the market.

## Figures and Tables

**Table 1:** Attributes and levels used in the discrete choice-based experiment design.

Attribute	Attribute Level
Increased content of ingredients	With an increased amount of vitamins and minerals
	With an increased amount of fiber
	With an increased amount of live bacteria cultures
	With an increased amount of cholesterol lowering ingredients
	With an increased amount of omega 3 acids
	With an increased amount of coenzyme Q10
	With an increased amount of ingredients positively affecting body shape, complexion, nails
	No information
Reduced content of ingredients	No added sugar
	Low energy content
	Low salt content
	Reduced sugar content
	No information
Additional claims	High nutritional value
	High health values
	High quality
	No information
Price	12 levels (from PLN * 1.00 to PLN 15.99)

* PLN = Polish new zloty (approx. 1 PLN = EUR 0.23).

**Table 2:** Example of the discrete choice-based experiment screen used in the study.

Option 1	Option 2	Option 3
Yoghurt		
with high fiber content	with an increased amount of live bacteria cultures	with an increased amount of cholesterol-lowering ingredients
with no added sugar	with law salt content	with reduced sugar content
with high health values	of high quality	of high nutritional value
PLN 12.99	PLN 3.59	PLN 2.29

**Table 3:** Socio-demographic profile of the total sample and the clusters identified (%) (*N* = 489).

Variable	Total Sample (*n* = 489)	Cluster 1 (*n* = 78, 16%)	Cluster 2 (*n* = 123, 25%)	Cluster 3 (*n* = 233, 48%)	Cluster 4 (*n* = 55, 11%)	Sig.
Gender						0.040
Female	59%	47%	58%	37%	56%	
Male	41%	53%	42%	63%	44%	
Education						0.484
Primary and junior high school	8%	13%	6%	6%	15%	
Vocational	29%	23%	32%	30%	29%	
Secondary	41%	38%	46%	43%	27%	
Higher	20%	26%	14%	19%	29%	
Refusal of answer	1%	0%	2%	2%	0%	
Age						0.008
34 and below	37%	28%	40%	43%	22%	
35–54	39%	40%	37%	37%	53%	
55–64	15%	18%	15%	14%	11%	
65 and over	9%	14%	8%	6%	15%	
Place of residence						0.000
Country	34%	37%	29%	29%	47%	
Up to 20,000	13%	13%	9%	15%	7%	
20,000–100,000	21%	26%	24%	20%	20%	
100,000–500,000	18%	15%	17%	24%	11%	
Over 500,000	14%	9%	21%	11%	15%	
Children						0.219
Yes	49%	46%	46%	48%	62%	
No	34%	28%	35%	38%	24%	
Refusal of answer	1%	1%	2%	1%	0%	
N.A.	16%	24%	18%	13%	15%	
Financial status						0.898
Sufficient budget without necessity to economise	10%	10%	11%	10%	11%	
We live frugally and have enough money to buy what we need	40%	47%	41%	38%	38%	
We live very frugally to save money for major purchases	28%	22%	24%	31%	31%	
We have enough money for the cheapest food or clothing	10%	9%	15%	9%	5%	
We have enough money for the cheapest food only, there is not enough money for clothing	2%	4%	0%	2%	4%	
There is not enough money even for the cheapest food or clothing	1%	0%	1%	1%	0%	
I don’t know/hard to say	7%	4%	7%	8%	9%	
Refusal of answer	2%	4%	2%	1%	2%	

**Table 4:** Clusters’ profile on attitudinal questionnaire referring to the importance of dairy products quality and openness to buy new products as well as changes in the market including dairy products (*N* = 489).

Statements	Total Sample	Cluster 1 QUALITY ORIENTED	Cluster 2 INVOLVED	Cluster 3 QUALITY ENTHUSIASTS	Cluster 4 NEUTRAL	Sig.
Quality matters to me while choosing a dairy products	5.96	6.28 a	5.40 b	6.15 a	5.96 a	0.000
I buy high-quality dairy products because they have a beneficial influence on the health of my children	5.11	4.91 a	4.93 a	5.23 a	5.24 a	0.375
I buy high-quality dairy products because they have a beneficial influence on my body shape	4.48	3.79 b	4.72 a	4.71 a	3.91 b	0.000
I buy high-quality dairy products only for those family members who have health issues	3.35	3.06 b,c	3.94 a	3.11 b,c	3.40 a,c	0.000
I am very particular about the new dairy products I will eat	4.79	4.49 a	4.63 a	4.98 a	4.76 a	0.139
Ethnic food deprived of dairy products looks too weird to eat (e.g., Asian cuisine)	4.58	4.88 a	4.46 a	4.49 a	4.76 a	0.954
If I do not know what is in a dairy product, I will not try it	4.54	4.71 a	4.37 a	4.51 a	4.82 a	0.514
New dairy products arouse my curiosity	4.39	3.04 b	4.78 a	5.00 a	2.23 b	0.000
I do not trust new foods ^1^	4.32	2.91 b	4.56 a	4.89 a	3.36 b	0.000
I like to buy new and various dairy products	4.26	1.71 c	4.92 a	5.24 a	2.24 b	0.000
I will eat virtually everything	4.16	3.69 b	4.52 a	4.30 a	3.38 b	0.000
At dinner parties, I will try new dishes based on dairy products	4.09	2.88 b	4.77 a	4.49 a	2.56 b	0.000
I like dairy products from cuisines of different countries	3.39	2.67 c	4.70 a	4.02 b	2.35 c	0.000
I constantly try new and varied dairy products	3.83	2.12 b	4.49 a	4.40 a	2.35 b	0.000
I am usually amongst the first ones to try new dairy products	3.74	1.95 b	4.59 a	4.27 a	2.11 b	0.000
I am afraid to eat dairy products I have never tried before	3.74	4.47 a	4.03 a	3.34 b,c	3.73 a,c	0.000
I know more than others about the latest dairy products	3.40	2.32 b	4.02 a	3.75 a	2.06 b	0.000
Among friends I am usually the first person to try new dairy products	3.30	1.81 b	4.20 a	3.64 a	2.02 b	0.000
I like to try ethnic restaurants (e.g., Asian cuisine)	3.27	2.51 b	4.26 a	3.19 b	2.49 b	0.000
I look for information about what new dairy products appear on the market	3.09	1.92 c	3.96 a	3.27 b	2.09 c	0.000

^1^ The item was reversed. a, b, c means with the same letter are not significantly different; ANOVA (Analysis of Variance) Tukey’s post hoc test.

**Table 5:** Clusters’ profile on attitudinal questionnaire referring to the importance of some attributes of yoghurt (*N* = 489).

Statements Referring to Attributes of Yoghurt that Is Usually Consumed	Total Sample	Cluster 1 QUALITY ORIENTED	Cluster 2 INVOLVED	Cluster 3 QUALITY ENTHUSIASTS	Cluster 4 NEUTRAL	Sig.
It was good for health	6.47	6.85 a	5.57 b	6.76 a	6.76 a	0.000
It was tasty	6.44	6.67 a	5.64 b	6.76 a	6.58 a	0.000
It was easily accessible	6.13	6.50 a	5.26 b	6.44 a	6.20 a	0.000
It had a pleasant smell	6.09	6.32 a	5.37 b	6.42 a	6.00 a	0.000
It was made in a traditional way	6.06	6.63 a	5.20 b	6.42 a	5.65 b	0.000
It came from farms where the producer pays special attention to the proper way of breeding animals	5.99	6.68 a	4.92 c	6.40 a	5.65 b	0.000
It was of Polish origin	5.97	6.55 a	5.05 b	6.33 a	5.69 c	0.000
It contained a lot of vitamins and minerals	5.91	6.27 a	5.09 b	6.31 a	5.55 b	0.000
It contained fiber	5.67	6.09 a	4.80 b	6.09 a	5.24 b	0.000
It was low in sugar	5.34	5.65 a	4.76 b	5.70 a	4.73 b	0.000
It was low in fat	5.10	5.28 a,c	4.73 b,c	5.43 a	4.25 b	0.000
It was low in calories	5.08	5.35 a	4.74 a,c	5.34 a	4.40 b,c	0.000
It contributed to maintaining a slim body	5.06	4.56 b	4.85 b	5.52 a	4.31 b	0.000
The packaging was appealing	4.95	4.55 b,c	4.69 c	5.48 a	3.85 b	0.000

a, b, c means with the same letter are not significantly different; ANOVA Tukey’s post hoc test.

**Table 6:** Profile on attitudinal questionnaire referring to purchase of various yoghurt types (*N* = 489).

Statements	Total Sample	Cluster 1 QUALITY ORIENTED	Cluster 2 INVOLVED	Cluster 3 QUALITY ENTHUSIASTS	Cluster 4 NEUTRAL	Sig.
With live bacteria cultures	5.89	6.19 a	5.07 c	6.23 a	5.87 b	0.000
Produced using raw materials from organic farms	5.81	6.36 a	4.98 b	6.17 a	5.36 b	0.000
With an increased level of substances beneficial to health	5.56	5.62 a,c	5.11 b,c	5.87 a	5.16 a,c	0.000
With reduced levels of some ingredients, e.g., salt or sugar, to prevent various diseases e.g., obesity, hypertension, diabetes	5.41	5.56 a,c	4.88 b	5.75 a	4.98 b,c	0.000
With higher levels of some vitamins and minerals to prevent nutritional deficiencies	5.40	5.41 b,c	4.98 b	5.73 a,c	4.89 b	0.000

a, b, c means with the same letter are not significantly different; ANOVA Tukey’s post hoc test.

**Table 7:** The part-worth utilities and relative importance of attributes for total sample and four identified clusters (*N* = 489).

Attribute	Attribute Level	Total Sample	Cluster 1 QUALITY ORIENTED	Cluster 2 INVOLVED	Cluster 3 QUALITY ENTHUSIASTS	Cluster 4 NEUTRAL	Sig.
Increased level of ingredients	Relative importance (%)	15.7	19.4	13.0	16.0	16.3	
	With an increased amount of vitamins and minerals	0.546	0.594	0.582	0.540	0.411	0.517
	With an increased amount of fiber	0.453	0.523	0.338	0.489	0.420	0.189
	With an increased number of live bacteria cultures	0.939	1.160	0.645	0.951	1.209	0.001
	With an increased amount of cholesterol lowering ingredients	0.133	0.188	0.062	0.160	0.118	0.751
	With an increased amount of omega3 acid	–0.232	–0.185	–0.292	–0.170	–0.412	0.427
	With an increased amount of coenzyme Q10	–0.774	–0.834	–0.552	–0.837	–0.860	0.073
	With an increased amount of ingredients that have a beneficial effect on the body shape, complexion and nails	–0.081	–0.360	0.191	–0.109	–0.149	0.005
	Lack of information	–0.985	–1.085	–0.973	–1.024	–0.737	0.262
Reduced level of ingredients	Relative importance (%)	5.4	8.5	4.2	6.2	3.6	
	No added sugar	0.323	0.497	0.147	0.382	0.241	0.032
	Low energy content	–0.338	–0.485	–0.206	–0.382	–0.221	0.008
	Low salt content	–0.038	–0.166	0.165	–0.085	–0.083	0.002
	With reduced sugar content	0.270	0.426	0.207	0.279	0.151	0.055
	Lack of information	–0.218	–0.272	–0.312	–0.195	–0.087	0.331
Additional information	Relative importance (%)	8.5	8.4	7.4	9.2	8.1	
	On high nutritional value	0.257	0.197	0.224	0.317	0.164	0.169
	On high health values	0.315	0.352	0.274	0.320	0.345	0.717
	On high quality	0.155	0.076	0.154	0.173	0.177	0.495
	Lack of information	–0.727	–0.625	–0.652	–0.811	–0.686	0.079
Price	Relative importance (%)	70.4	63.7	75.4	68.7	71.9	
	PLN * 1.00	5.064	4.251	5.540	4.978	5.441	0.301
	PLN 1.29	4.547	3.805	5.000	4.477	4.822	0.230
	PLN 1.69	3.670	3.147	4.034	3.586	3.882	0.140
	PLN 2.19	3.118	2.661	3.436	3.085	3.126	0.094
	PLN 2.79	1.634	1.549	1.721	1.617	1.594	0.580
	PLN 3.59	–0.535	–0.316	–0.705	–0.481	–0.663	0.089
	PLN 4.59	–1.568	–1.249	–1.766	–1.540	–1.667	0.165
	PLN 5.89	–2.502	–2.099	–2.699	–2.490	–2.625	0.353
	PLN 7.59	–3.108	–2.691	–3.392	–3.059	–3.220	0.233
	PLN 9.79	–3.288	–2.879	–3.568	–3.238	–3.403	0.198
	PLN 12.99	–3.468	–3.046	–3.754	–3.417	–3.598	0.191
	PLN 15.99	–3.564	–3.133	–3.848	–3.518	–3.687	0.164

* PLN = Polish new zloty (approx. 1 PLN = EUR 0.23).
